# What Matters Most to Veterans When Deciding to Use Technology for Health: Cross-Sectional Analysis of a National Survey

**DOI:** 10.2196/77113

**Published:** 2025-08-07

**Authors:** Bella Etingen, Bridget M Smith, Stephanie L Shimada, Stephanie A Robinson, Robin T Higashi, Ndindam Ndiwane, Kathleen L Frisbee, Jessica M Lipschitz, Eric Richardson, Dawn Irvin, Timothy P Hogan

**Affiliations:** 1 eHealth Partnered Evaluation Initiative VA Bedford Healthcare System Bedford, MA United States; 2 Research and Development Service Dallas VA Medical Center Dallas, TX United States; 3 Peter O’Donnell Jr. School of Public Health The University of Texas Southwestern Medical Center Dallas, TX United States; 4 Center of Innovation for Complex Chronic Healthcare Edward Hines, Jr. VA Hospital Hines, IL United States; 5 Feinberg School of Medicine Northwestern University Chicago, IL United States; 6 Center for Health Optimization and Implementation Research VA Bedford Healthcare System Bedford, MA United States; 7 Department of Health Law, Policy, and Management School of Public Health Boston University Boston, MA United States; 8 Department of Population and Quantitative Health Sciences University of Massachusetts Chan Medical School Worcester, MA United States; 9 The Pulmonary Center School of Medicine Boston University Boston, MA United States; 10 Office of Connected Care Veterans Health Administration Washington, DC United States; 11 Brigham and Women's Hospital Boston, MA United States; 12 Department of Psychiatry Harvard Medical School Boston, MA United States; 13 Center for Health Optimization and Implementation Research VA Boston Healthcare System Boston, MA United States

**Keywords:** digital health technology, implementation, mobile health apps, patient preferences, veterans

## Abstract

**Background:**

There is an increasingly diverse range of mobile apps and digital health devices available to help patients manage their health. Despite evidence for the effectiveness of such technologies, their potential has not been fully realized because adoption remains low. Such limited uptake can have direct implications for the intended benefits of these technologies.

**Objective:**

This study aimed to understand what matters most to US military veterans when deciding whether to use digital health technologies (DHTs) such as mobile health apps or devices to manage their health and compare these factors between veterans with and without prevalent chronic physical and mental health conditions.

**Methods:**

We conducted a cross-sectional analysis of survey data collected from a national sample of veterans who receive care from the Veterans Health Administration (VHA), which was predominantly gathered as part of the last wave of a larger longitudinal data collection effort.

**Results:**

Among respondents (n=857), 86.7% (736/849) reported currently using or having previously used ≥1 devices to manage their health, and 78.4% (639/815) also reported using either VHA or non-VHA health apps. Considerations most frequently endorsed as “very important” by veterans when deciding whether to use DHTs included receiving secure messages from their health care team about DHTs, knowing data from DHTs would be used to inform their care, and receiving recommendations from providers to use DHTs. Conversely, considerations most frequently endorsed as “not at all important” included seeing information about DHTs on social media, having community support to use DHTs, and receiving encouragement from peers to use DHTs. Considerations did not significantly differ between veterans with or without prevalent chronic health conditions; however, a greater proportion of veterans with prevalent mental health conditions reported the following considerations to be “very important:” seeing information about DHTs on social media, having community support to use DHTs, having other veterans encourage DHT use, and having help from family, friends, or other important people to use DHTs.

**Conclusions:**

Understanding what matters most to patients when they are deciding to adopt a technology for their health can, and should, inform implementation strategies and other approaches to enhance health-related technology use. Our results suggest that, for veterans, recommendations from health care team members and knowing that the data from DHTs will be used in clinical care are more important than information from social media, community sources, or peers when deciding to use DHTs, although perceptions of importance regarding the latter may differ among patients with different conditions. Our findings suggest that communication from health care team members to patients, perhaps either in-person or electronically, could help encourage DHT adoption and use.

## Introduction

Mobile apps to support health management are proliferating and can support condition self-management, health behavior tracking, and health education [1-4]. Mobile health apps can be accessed and used on or with various technology platforms and digital health–related devices, including smartphones, tablet computers, and sensor technologies like smart watches. We refer to such mobile health apps and digital health–related devices as digital health technologies (DHTs).

Evidence suggests that DHT use can positively affect health behaviors and outcomes [5-9]. Accordingly, the Veterans Health Administration (VHA), the largest integrated health care system in the United States, is championing incorporation of DHTs into care. However, adoption and use of DHTs remain limited, which are among the most significant problems facing DHTs [10-17]. Prominent theoretical frameworks posit various constructs that can influence uptake of different technologies, including, but not limited to, perceptions of ease of use, usefulness, the opinions of others, and existing support for technology use, although the relative importance of such factors varies in different contexts [18-20].

For DHTs to realize their potential, efforts to bolster adoption are needed. In implementation research, there is increasing recognition of the importance of stakeholder-informed strategies to support intervention adoption [21-24], that is, it is key to understand what stakeholders (in this case, patients who use technology for health) think the most effective ways are for promoting adoption and use of interventions and to design and tailor implementation strategies accordingly [25].

Our objective in this evaluation was to understand what matters most to veterans who are known technology users when deciding whether to use DHTs and to compare perceptions between those with and without prevalent chronic physical and mental health conditions to inform the development of future implementation strategies for DHTs.

## Methods

### Design

In partnership with researchers from the VHA’s Quality Enhancement Research Initiative Program, the VHA Office of Connected Care, responsible for the VHA’s digital health strategy, launched the Veterans Engagement with Technology Collaborative (VET-C) cohort in 2017. A longitudinal data collection effort, the VET-C cohort combines veteran survey data collected over 3 timepoints with VHA administrative records [26]. The goal of the cohort is to support veteran engagement in the evaluation of VHA patient-facing technologies [26]. This manuscript reports the results of a cross-sectional analysis of data gathered predominantly as part of the third round of data collection efforts from the VET-C cohort to understand perspectives on what is most important to veterans when deciding whether to use DHTs, with the goal to inform implementation strategies that the VHA can leverage to support DHT adoption.

### Participants

The VET-C cohort consists of a national sample of veterans who received care from 14 geographically distinct VHA facilities. These sites were selected purposefully to represent VHA facilities with high use of secure messaging supported by My HealtheVet (the VHA’s web-based patient portal), diverse segments of the veteran population, hubs for VHA research, and experience with technology rollouts. Data collection did not focus on a specific clinical setting. Veterans invited to the cohort were active users of VHA health care and technology users, defined as owning a cellular phone and having sent ≥5 secure messages through My HealtheVet in the previous year [26,27].

VET-C surveys were collected between 2017-2018, 2019-2020, and 2021-2022. As noted above, data for the variables reported in this analysis were primarily gathered in the third round of VET-C data collection from veterans who had responded to the first 2 rounds of surveys. Information about the first 2 rounds of VET-C survey data collection is published elsewhere [26-28].

### Data Collection

Our recruitment procedures comprised several steps. Veterans were first mailed a copy of the third round VET-C survey along with a cover letter and postage-paid reply envelope. A follow-up mailing was then sent to nonrespondents approximately 4 weeks after the initial mailing to facilitate response. The survey was designed iteratively based on veteran feedback and Office of Connected Care leadership input and included a combination of validated items and customized questions.

### Measures

Veterans were asked to report their sex, age, race and ethnicity, highest level of education, relationship status, self-perceived physical and mental health status [29], extent of health care received through the VHA (compared to community settings), and typical travel time (in minutes) from home to their VHA primary care doctor.

We further asked veterans to report technology access and ownership, technology use [30], and whether they are mobile health app users and health-related digital device users. We also asked veterans to report how easy it is for them to communicate with their VHA health care team.

We collected information on perceptions of what is important when deciding whether to use technology for health by asking veterans to rate various considerations as “very important,” “somewhat important,” or “not at all important” when deciding whether to use DHTs to help them manage their health. Response options included considerations at multiple levels, including those related to the health care system, the community, the patient themselves, and an open-ended “other” response option where respondents could list and rate considerations not otherwise included among the closed-ended response options.

We supplemented survey data with administrative data to identify chronic physical and mental health conditions from the prior 5 years that are prevalent among veterans and to fill in missing demographic data as necessary. We also used administrative data to calculate a prior-year comorbidity index score using the hierarchical condition community score [31,32]. The hierarchical condition community score factors in a person’s diagnoses, eligibility for Medicare and Medicaid services, sex, and age and commonly ranges from 0.9-1.7, with scores greater than 1 often interpreted as indicating poorer health [31-33].

### Analyses

We used descriptive statistics to characterize survey data, and chi-square analyses to compare perceptions of what veterans consider important when deciding whether to use technology for health, comparing those with and without prevalent chronic health conditions (ie, diabetes, hypertension, and chronic kidney disease) and prevalent mental health conditions (ie, depression, anxiety, and posttraumatic stress disorder). Analyses were conducted in Stata MP (version 14.2; StataCorp).

### Ethical Considerations

This work was reviewed by relevant VHA Institutional Review Boards and designated as program evaluation for quality improvement purposes, exempting it from further oversight (Program Guide 1200.21) [34]. Returning a survey was considered consent to participate. Data were deidentified and labeled with a unique ID number. Veterans were not compensated for their participation.

## Results

### Response Rate

We invited 1373 veterans to complete a third-round survey. We removed 15 veterans from the denominator because their surveys were undeliverable or they had passed away. A total of 858 (63.2%) veterans returned a survey. We removed 1 veteran with unavailable health condition data from our analytic cohort.

### Sample Description

As summarized in Tables 1 and 2, survey respondents were predominantly men (750/857, 87.5%), White (762/857, 88.9%), and older than 65 years of age (602/857, 70.2%). Over half (452/853, 53%) considered themselves early technology adopters. Most (736/849, 86.7%) reported currently using or previously having used ≥1 health-related digital devices and currently or previously using either VHA or non-VHA mobile health apps (639/815, 78.4%).

**Table 1:** Demographic of veteran respondents to the Veterans Engagement with Technology Collaborative cohort survey.

Demographic			n (%)
**Age (n=857)**			
	Aged over 65 years	602 (70.2)	
	Aged 65 years or younger	255 (29.8)	
**Sex (n=857)**			
	Men	750 (87.5)	
	Women	107 (12.5)	
**Race (n=857)**			
	White	762 (88.9)	
	Black	55 (6.4)	
	Other	40 (4.7)	
**Ethnicity (n=857)**			
	Non-Hispanic	830 (96.9)	
	Hispanic	27 (3.3)	
**Relationship status (n=826)**			
	In a relationship	610 (73.8)	
	Not in a relationship	216 (26.2)	
**Education status (n=851)**			
	At least some college or vocational school	745 (87.5)	
	Less than some college or vocational school	106 (12.5)	
**Socioeconomic status (n=775)**			
	Not very hard to pay for basics^a^	586 (75.6)	
	Hard to pay for basics	189 (24.4)	
**Location of medical care (n=856)**			
	Mostly receives medical care in the VHA^b^	616 (72)	
	Does not mostly receive medical care in the VHA	240 (28)	
**Time to VHA primary care doctor’s office (n=856)**			
	<60 min	726 (84.8)	
	>60 min	130 (15.2)	
**Perceived physical health status (n=853)**			
	Good or very good or excellent	596 (69.9)	
	Fair or poor	257 (30.1)	
**Perceived mental health status (n=855)**			
	Good or very good or excellent	694 (81.2)	
	Fair or poor	161 (18.8)	
**Ease of communication (n=850)**			
	Very easy or easy	664 (78.1)	
	Very difficult or difficult or neutral	186 (21.9)	
Hierarchical condition community score (n=857), mean (SD)			1.7 (1.3)
**Technology ownership** **(n=849)**			
	Desktop or laptop computer	793 (93.4)	
	Smartphone	761 (89.6)	
	Tablet computer	448 (52.8)	
**Technology adoption (n=853)**			
	Early technology adopter	452 (53)	
	Not an early technology adopter	401 (47)	
**Comfort using computers (n=852)**			
	Very comfortable or confident using computers	759 (89.1)	
	Not very comfortable or confident using computers	93 (10.9)	
**Self-reported mobile health app user?** **(n=815)**			
	Yes	639 (78.4)	
	No	176 (21.6)	
**Self-reported health-related digital device user?** **(n=849)**			
	Yes	736 (86.7)	
	No	113 (13.3)	

^a^Basics refers to essential needs like food and heating and cooling.

^b^VHA: Veterans Health Administration.

**Table 2:** Health conditions of veteran respondents to the Veterans Engagement with Technology Collaborative cohort survey. Health conditions from only the prior 5 years are included.

Health condition (n=857)	n (%)
Hypertension	667 (77.8)
Osteoarthritis	453 (52.9)
Anxiety disorders	404 (47.1)
Diabetes	377 (44)
Depression	327 (38.2)
Chronic kidney disease	257 (30)
Ischemic heart disease	256 (29.9)
Asthma	204 (23.8)
Posttraumatic stress disorder	251 (29.3)
Chronic obstructive pulmonary disease	158 (18.4)
Peripheral vascular disease	136 (15.9)
Atrial fibrillation	130 (15.2)
Heart failure	92 (10.7)
Prostate cancer	65 (7.6)
Stroke	56 (6.5)
Traumatic brain injury	26 (3)
Acute myocardial infarction	25 (2.9)
Lung cancer	20 (2.3)
Colorectal cancer	13 (1.5)

### Considerations When Deciding Whether to Use DHTs

Considerations most frequently endorsed as “very important” included receiving secure messages through the VHA’s My HealtheVet web-based patient portal from one’s health care team about DHTs (561/766, 73.2%), knowing that one’s health care team will use the information entered into the DHTs to inform one’s care (427/756, 56.5%), having one’s provider recommend DHTs (402/764, 52.6%), having somewhere to get help with issues using DHTs (389/760, 51.2%), and having other members of one’s health care team recommend DHTs (345/760, 45.4%). These considerations did not significantly differ between veterans with or without prevalent chronic health conditions or prevalent mental health conditions.

The considerations most frequently endorsed as “not at all important” by veterans included the following: seeing information about DHTs on social media (533/756, 70.5%); having community support through Veteran Service Organizations, churches, libraries, or other organizations (501/754, 66.4); having other veterans encourage DHT use (430/758, 56.7%); having help from family, friends, or other important people to use DHTs (315/759, 41.6%); and receiving printed information about DHTs from the VHA (201/758, 26.5%). These considerations did not significantly differ between veterans with or without prevalent chronic health conditions. However, a greater proportion of veterans with (compared to without) prevalent mental health conditions reported the following considerations to be “very important”: seeing information about DHTs on social media (those with mental health conditions: 42/428, 9.8%; those without mental health conditions: 19/328, 5.8%; *χ*^2^_2_=6.2; *P*=.05); having community support through Veteran Service Organizations, churches, libraries, or other organizations to use DHTs (with: 56/427, 13.1%; without: 25/327, 7.6%; *χ*^2^_2_=7.9; *P*=.02); having other veterans encourage DHT use (with: 70/429, 16.3%; without: 24/329, 7.3%; *χ*^2^_2_=15.9; *P*<.001); and having help from family, friends, or other important people to use DHTs (with: 106/431, 24.6%; without: 58/328, 17.7%; *χ*^2^_2_=6.4; *P*=.04). Please see Table 3 for more details.

**Table 3:** Perceptions of importance regarding factors that could impact decisions about the use of digital health technologies^a^ among veteran respondents to the Veterans Engagement with Technology Collaborative cohort survey and comparisons of perceptions among veterans with and without prevalent chronic health conditions and prevalent mental health conditions (n=857).

Factor			Overall, n/N (%)		Respondent has ≥1 chronic health conditions^b^ n/N (%)								Respondent has ≥1 mental health conditions^c^ n/N (%)					
					Yes (713/857)		No (144/857)		*P*			Yes (481/857)			No (376/857)			*P*
**Receiving MyHealtheVet secure messages from my VHA^d^ health care team about them**									.09									.26
	Very important	561/766 (73.2)		474/641 (73.9)		87/125 (69.6)					325/435 (74.7)			236/331 (71.3)				
	Somewhat important	158/766 (20.6)		133/641 (20.7)		25/125 (20)					81/435 (18.6)			77/331 (23.3)				
	Not at all important	47/766 (6.1)		34/641 (5.3)		13/125 (10.4)					29/435 (6.7)			18/331 (5.4)				
**Knowing that my VHA health care team members will use the information that I enter into them to inform my health care**									.56									.19
	Very important	427/756 (56.5)		355/631 (56.3)		72/125 (57.6)					254/428 (59.3)			173/328 (52.7)				
	Somewhat important	248/756 (32.8)		205/631 (32.5)		43/125 (34.4)					132/428 (30.8)			116/328 (35.4)				
	Not at all important	81/756 (10.7)		71/631 (11.3)		10/125 (8)					42/428 (9.8)			39/328 (11.9)				
**Having my VHA doctor(s) recommend them to me**									.24									.88
	Very important	402/764 (52.6)		344/638 (53.9)		58/126 (46)					230/433 (53.1)			172/331 (52)				
	Somewhat important	274/764 (35.9)		221/638 (34.6)		53/126 (42.1)					152/433 (35.1)			122/331 (36.9)				
	Not at all important	88/764 (11.5)		73/638 (11.4)		15/126 (11.9)					51/433 (11.8)			37/331 (11.2)				
**Having somewhere to get help if I am having issues using them**									.82									.18
	Very important	389/760 (51.2)		322/634 (50.8)		67/126 (53.2)					232/431 (53.8)			157/329 (47.7)				
	Somewhat important	262/760 (34.5)		219/634 (34.5)		43/126 (34.1)					137/431 (31.8)			125/329 (38)				
	Not at all important	109/760 (14.3)		93/634 (14.7)		16/126 (12.7)					62/431 (14.4)			47/329 (14.3)				
**Having other members of my VHA clinical team recommend them to me**									.11									.39
	Very important	345/760 (45.4)		297/635 (46.8)		48/125 (38.4)					203/429 (47.3)			142/331 (42.9)				
	Somewhat important	307/760 (40.4)		246/635 (38.7)		61/125 (48.8)					170/429 (39.6)			137/331 (41.4)				
	Not at all important	108/760 (14.2)		92/635 (14.5)		16/125 (12.8)					56/429 (13.1)			52/331 (15.7)				
**Having a video tutorial about how to use them**									.57									.81
	Very important	255/758 (33.6)		208/633 (32.9)		47/125 (37.6)					146/429 (34)			109/329 (33.1)				
	Somewhat important	315/758 (41.6)		265/633 (41.9)		50/125 (40)					174/429 (40.6)			141/329 (42.9)				
	Not at all important	188/758 (24.8)		160/633 (25.3)		28/125 (22.4)					109/429 (25.4)			79/329 (24)				
**Having someone available to help me learn how to use them through in-person trainings**									.43									.40
	Very important	241/758 (31.8)		202/633 (31.9)		39/125 (31.2)					144/429 (33.6)			97/329 (29.5)				
	Somewhat important	273/758 (36)		233/633 (36.8)		40/125 (32)					147/429 (34.3)			126/329 (38.3)				
	Not at all important	244/758 (32.2)		198/633 (31.3)		46/125 (36.8)					138/429 (32.2)			106/329 (32.2)				
**Receiving printed information about them from the VHA**									.58									.37
	Very important	231/758 (30.5)		197/632 (31.2)		34/126 (27)					139/427 (32.6)			92/331 (27.8)				
	Somewhat important	326/758 (43)		271/632 (42.9)		55/126 (43.7)					179/427 (41.9)			147/331 (44.4)				
	Not at all important	201/758 (26.5)		164/632 (25.9)		37/126 (29.4)					109/427 (25.5)			92/331 (27.8)				
**Having help from family, friends, or other important people in my life to use them**									.24									.04
	Very important	164/759 (21.6)		143/633 (22.6)		21/126 (16.7)					106/431 (24.6)			58/328 (17.7)				
	Somewhat important	279/759 (36.8)		226/633 (35.7)		53/126 (42.1)					146/431 (33.9)			133/328 (40.5)				
	Not at all important	316/759 (41.6)		264/633 (41.7)		52/126 (41.3)					179/431 (41.5)			137/328 (41.8)				
**Having other veterans encourage me to use them**									.69									<.001
	Very important	94/758 (12.4)		77/634 (12.1)		17/124 (13.7)					70/429 (16.3)			24/329 (7.3)				
	Somewhat important	234/758 (30.9)		193/634 (30.4)		41/124 (33.1)					135/429 (31.5)			99/329 (30.1)				
	Not at all important	430/758 (56.7)		364/634 (57.4)		66/124 (53.2)					224/429 (52.2)			206/329 (62.6)				
**Having support in the community through my VSO^e^, church, library, or other such organizations for using them**									.37									.02
	Very important	81/754 (10.7)		64/630 (10.2)		17/124 (13.7)					56/427 (13.1)			25/327 (7.6)				
	Somewhat important	172/754 (22.8)		148/630 (23.5)		24/124 (19.4)					103/427 (24.1)			69/327 (21.1)				
	Not at all important	501/754 (66.4)		418/630 (66.3)		83/124 (66.9)					268/427 (62.8)			233/327 (71.3)				
**Seeing information about them on social media**									.52									.05
	Very important	61/756 (8.1)		48/631 (7.6)		13/125 (10.4)					42/428 (9.8)			19/328 (5.8)				
	Somewhat important	162/756 (21.4)		134/631 (21.2)		28/125 (22.4)					98/428 (22.9)			64/328 (19.5)				
	Not at all important	533/756 (70.5)		449/631 (71.2)		84/125 (67.2)					288/428 (67.3)			245/328 (74.7)				
**Other**									.67^f^							.34^f^		
	Very important	12/76 (15.8)		10/68 (14.7)		2/8 (25)					8/40 (20)			4/36 (11.1)				
	Somewhat important	7/76 (9.2)		7/68 (10.3)		0/8 (0)					5/40 (12.5)			2/36 (5.6)				
	Not at all important	57/76 (75)		51/68 (75)		6/8 (75)					27/40 (67.5)			30/36 (83.3)				

^a^VHA mobile health apps, non-VHA mobile health apps, or devices.

^b^Hypertension, diabetes, or chronic kidney disease.

^c^Anxiety, depression, or posttraumatic stress disorder.

^d^VHA: Veterans Health Administration.

^e^VSO: Veteran Service Organization.

^f^Fisher exact test *P* value.

## Discussion

### Principal Findings

Understanding what matters most to patients when deciding to use DHTs can inform the development of strategies to support their adoption and use. For veterans who receive VHA care and are known technology users, our results suggest that the most important considerations are tied to the health care system and include receiving encouragement from health care team members to use DHTs, knowing DHT data will be used in care, and having help available when using DHTs. Conversely, our results suggest that the least important considerations for veterans who use VHA care and are known technology users when deciding to use DHTs are rooted outside the health care system, including social media and encouragement and support from peers or other community members, although the veterans in our sample with prevalent mental health conditions did place more importance on these considerations. These insights can be leveraged to promote different technology platforms as part of health care services and inform efforts to ensure that implementation strategies deployed align with patient preferences and values. They also reflect the relative importance in this context of different theoretical constructs posited to influence the adoption of new technologies, including sources of social influence and the perceptions of others deemed important to an individual [18,19].

From our findings, outreach and recommendations from care team members were most frequently endorsed as very important to decisions about DHT use. These findings are consistent with previous literature that highlights the importance of health care team member encouragement to veteran use of technologies for health [12,14,35,36], and similar findings have also emerged in other published studies of other patient populations [37,38]. This trend suggests that implementation strategies intended to bolster veteran DHT adoption and use must reflect care team member involvement to be optimally impactful. This could include emphasizing care team member endorsement of specific technologies and their potential value to veterans or affirmation that care team members will use the data that veterans gather and share through these technologies to inform clinical practice, a consideration that has also been highlighted as important in literature about nonveteran populations [39].

Uniquely, our findings highlight different approaches that can be useful for outreach and recommendations and how such approaches may have differential impacts among segments of patient populations. In this sample of veterans who are known technology users, receiving secure messages about DHTs through the VHA’s web-based patient portal was the consideration most frequently endorsed as very important when deciding to use DHTs. The VHA already uses its web-based patient portal to disseminate health promotion information, including information about new technologies [12]. Using the messaging feature of a web-based patient portal may be an efficient, low-cost way for health care teams to proactively communicate with patients about technologies they think could be helpful to them.

Beyond health care team–related factors, the availability of technical support was the second most frequent consideration endorsed as very important when deciding to use DHTs. Previously published reviews have also identified difficulties using technology and tech support availability as factors influencing patient use [40,41]. Although our survey did not ask veterans whether they thought such support should be offered by the VHA, we see such help and support as services that the VHA could provide to address this need. Such services are evident in recent VHA initiatives like Veteran Health Resource Centers, which provide hands-on support within VHA facilities to veterans, their informal caregivers, and staff who are interested in using VHA technology platforms [42].

Unlike previously published studies suggesting that leveraging communities, peers, and social media may facilitate the adoption of technology for health in certain contexts [43-45], the veterans in our sample less frequently endorsed peer and community support and information shared through social media as important considerations. Although peer-, community-, and social media–based resources may represent efficient and low-cost approaches to facilitating DHT use, health care systems should work to understand patient preferences and perspectives before relying too heavily on such approaches, which may be best suited for use among certain segments of their patient populations. Patients with mental health conditions may be one such example, where strategies grounded in community and peer support are well-positioned to enhance reach and engagement with DHTs.

### Limitations

The veterans in the VET-C cohort are known technology users. As such, our results may not generalize to the overall veteran population or other patient populations. This sample characteristic may also explain why receiving secure messages from one’s health care team through the VHA’s web-based patient portal was deemed such an important consideration. However, this finding parallels other findings regarding the importance that veterans place on health care team member recommendations when deciding whether to use DHTs [14,35]. Moreover, data from our sample of known technology users may not address considerations important to veterans who minimally or do not use technology when deciding to use DHTs. We must guard against overgeneralizing these findings so as not to inadvertently overlook the needs of patients for whom accessibility of care, and by extension DHTs, are formidable barriers. These groups may require alternative implementation strategies that more adequately reflect their situation [46,47]. Potentially high-impact future work could include replicating a survey similar to the one reported here with subsets of the veteran population who lack both ready access to DHTs and health care services. Cross-sectional survey data may also be influenced by recall, response, and social desirability biases. Finally, we cannot infer how our results may translate to actual technology adoption and use. Additional work is needed to develop and rigorously test implementation strategies to provide such insights.

### Conclusions

Understanding what matters most to patients when deciding to use DHTs can inform strategies to enhance DHT use. This analysis represents an additional step towards foregrounding the perspective of patients as key stakeholders when designing implementation strategies to bolster adoption of health-related technology. Recommendations from health care team members and knowing their data will be used by their care team members in clinical care are more important considerations for veterans who use technology for health than information they may encounter through social media, community sources, or peers when deciding whether to adopt DHTs, although perceptions of importance regarding the latter may differ among patients with different conditions. Taken together, our results suggest that communication from health care team members to patients, perhaps either in-person or electronically, may be beneficial in many cases to promote DHT adoption and use. Future work should also examine these issues among veterans who may have a strong interest in using DHTs but are not yet DHT users and face various barriers to their adoption.

## Abbreviations

DHT: digital health technology
VET-C: Veterans Engagement with Technology Collaborative
VHA: Veterans Health Administration

## Data Availability

The data used for this analysis are not permitted to leave the VHA firewall without a data use agreement. This limitation is consistent with other work based on VHA data. However, VHA data are made freely available to investigators behind the Department of Veterans Affairs’ firewall with appropriate documentation.
